# A Drying-Rewetting Cycle Imposes More Important Shifts on Soil Microbial Communities than Does Reduced Precipitation

**DOI:** 10.1128/msystems.00247-22

**Published:** 2022-06-28

**Authors:** Xiao-Bo Wang, Hamed Azarbad, Laura Leclerc, Jessica Dozois, Eugenie Mukula, Étienne Yergeau

**Affiliations:** a State Key Laboratory of Grassland Agroecosystems, Center for Grassland Microbiome, Lanzhou University, Lanzhou, People’s Republic of China; b College of Pastoral, Agriculture Science and Technology, Lanzhou University, Lanzhou, People’s Republic of China; c Centre Armand-Frappier Santé Biotechnologie, Institut National de la Recherche Scientifique, Laval, Québec, Canada; d Department of Biology, Evolutionary Ecology of Plants, Philipps University Marburg, Marburg, Germany; e Erguna Forest-Steppe Ecotone Research Station, Institute of Applied Ecology, Chinese Academy of Sciences, Shenyang, People’s Republic of China; Michigan State University

**Keywords:** soil microbes, bacterial and fungal diversity, community assembly, rainfall, dry-rewet events, wheat cultivar, agroecosystems, global change, microbial diversity, precipitation

## Abstract

Global changes will result in altered precipitation patterns, among which the increasing frequency of drought events has the highest deleterious potential for agriculture. Soil microbes have shown some promise to help crops adapt to drought events, but it is uncertain how crop-associated microorganisms will respond to altered precipitation patterns. To investigate this matter, we conducted a field experiment where we seeded two wheat cultivars (one resistant to water stress and the other sensitive) that were subjected to four precipitation exclusion (PE) regimes (0%, 25%, 50%, and 75% exclusion). These cultivars were sampled seven times (every 2 weeks, from May to August) within one growing season to investigate short-term microbiome responses to altered precipitation regimes and seasonality using 16S rRNA gene and internal transcribed spacer (ITS) region amplicon sequencing. One of the most striking features of the data set was the dramatic shift in microbial community diversity, structure, and composition together with a doubling of the relative abundance of the archaeal ammonia oxidizer genus *Nitrososphaera* following an important drying-rewetting event. Comparatively small but significant effects of PE and wheat cultivar on microbial community diversity, composition, and structure were observed. Taken together, our results demonstrate an uneven response of microbial taxa to decreasing soil water content, which was dwarfed by drying-rewetting events, to which soil bacteria and archaea were more sensitive than fungi. Importantly, our study showed that an increase in drying-rewetting cycles will cause larger shifts in soil microbial communities than a decrease in total precipitation, suggesting that under climate changes, the distribution of precipitation will be more important than small variations in the total quantity of precipitation.

**IMPORTANCE** Climate change will have a profound effect on the precipitation patterns of global terrestrial ecosystems. Seasonal and interannual uneven distributions of precipitation will lead to increasing frequencies and intensities of extreme drought and rainfall events, which will affect crop productivity and nutrient contents in various agroecosystems. However, we still lack knowledge about the responses of soil microbial communities to reduced precipitation and drying-rewetting events in agroecosystems. Our results demonstrated an uneven response of the soil microbiome and a dramatic shift in microbial community diversity and structure to a significant drying-rewetting event with a large increase in the relative abundance of archaeal ammonia oxidizers. These findings highlight the larger importance of rewetting of dry soils on microbial communities, as compared to decreased precipitation, with potential for changes in the soil nitrogen cycling.

## INTRODUCTION

Precipitation patterns changed during the past few decades and are projected to change even further in the coming decades, with predicted higher frequencies and intensities of extreme drought and rainfall events (1, 2). The inter- and intra-annual variability of precipitation (3, 4), in addition to the uneven seasonal distribution of precipitation (5–7), will be further intensified by these altered precipitation patterns. Agroecosystems are particularly susceptible to drying-rewetting cycles caused by uneven precipitation events and their seasonal distribution. According to *Canada’s Changing Climate Report* (CCCR) (8), the province of Québec, Canada, will experience significant variations in monthly rainfall. Such variations in precipitation patterns could profoundly impact soil biotic and abiotic processes and, consequently, crop yields (9). However, the effects of changing precipitation patterns on the soil microbial communities of agroecosystems are not well understood, even though shifts in microbial communities could compound or cancel the direct effects of water stress on crops (10).

Soil microorganisms are key drivers of biogeochemical cycling in ecosystems and are driven by spatial and temporal variations in precipitation/water availability (11–13). Frequent drying-rewetting events brought about by the uneven distribution of precipitation can have important effects on microbial community structure, composition, and activities, with consequences for microbe-mediated soil biogeochemical processes (14). Rewetting of dry soil has been found to result in a large pulse of soil respiration (15, 16), enhanced substrate decomposition rates (17), and increased nitrogen (N) mineralization (18) and leaching (19, 20). Under these conditions, understanding the responses of soil microbial communities to changing precipitation regimes over time is extremely important to model ecosystem carbon (C) balance (21–23) and to predict changes in ecosystem processes (24) and the consequences of global climate change on ecosystem function (25, 26).

A large number of studies have demonstrated that climate events, such as precipitation intensity and seasonality or drying-rewetting, can have a positive or negative impact on soil microorganisms via stimulating or suppressing their growth and activity (27–29). Such an effect can persist for a short or a long time (30) and may result in a legacy effect on the soil microbial communities (31, 32). For example, microbes that have been subjected to low water availability for a long time may tend to be stressed during sudden precipitation or rewetting events because of rapid changes in osmotic pressure (33, 34) or may be better equipped to cope with this sudden change due to soil legacy effects (35) that constrain their responses to further water stresses (36). Increased soil water availability can also reduce the oxygen concentration in soils and suppress microbial activity (28). Previous studies focusing on the short-term effects of drying-rewetting have shown that the rapid rewetting of dry soil can stimulate soil microorganisms and induce a pulse in nutrient mineralization (e.g., C and N mineralization rates) for a few days (37, 38). Yet soil C mineralization rates were shown to decrease significantly over time in soils subjected to repeated drying-rewetting (39). Since nitrification is generally constrained by NH_4_^+^ diffusion in dry soils, rapid rewetting of dry soils will also lead to a flush of available N and the stimulation of nitrifiers (40). This will result in ecosystem nitrogen saturation, even in nitrogen-limited ecosystems (41). These changes in microbial respiration and mineralization are accompanied by shifts in microbial community diversity, composition, structure, and function (9, 42) and affect crop nutrient acquisition, growth, and productivity (43). Nevertheless, to date, the effects of soil water content and drying-rewetting cycles on the soil-associated microbial communities of agroecosystems were never contrasted.

The effects of precipitation variation on microbial communities are dependent upon multiple factors, such as taxonomy, phylogeny, and physiological characteristics of the individual microorganisms making up the community. Due to potential differences in phylogenetic relatedness (44), body size (45), and life strategies (46), different microbial groups will respond differently to fluctuating precipitation (47, 48). Shifts in precipitation can result in differences in community fitness under drought or rewetting stress, in such a way that certain environmental conditions favoring some taxa or functional groups would likely be adverse to others (14, 49). In this case, certain abundant taxa/populations in a community may become rare and even extinct as drought or rewetting stress goes beyond their physiological tolerance limit (50). Selection for taxa resulting from physiological stress and trade-offs will thus result in shifts in microbial community assembly toward a community that better withstands the new environmental conditions than prior communities (51, 52).

Water availability also plays an essential role in structuring plant communities at short and long temporal scales (53). Plants have a wide range of strategies to face variations in water availability and the associated stresses via changing their leaf and root traits, root exudates, and phenology (54). These physiological responses to water stress can certainly affect litter production, exudate compositions, and, consequently, the amount and quality of C available for microorganisms (55). As a result, the responses of soil microorganisms to water stress not only depend on the differences in physiological attributes between them but also can be indirectly affected by the response of plants (56–58).

In addition, fungi and bacteria have different strategies to cope with water stress (59, 60). It has been reported that fungi in soils are able to use their hyphae to transfer moisture from water-filled micropores (61), while bacteria need water films on the soil surface for dispersion and substrate diffusion (62). This difference in adaptive strategies allows soil fungi to regulate osmotic stress by their extensive hyphal networks to efficiently transfer water and nutrients in a dry environment (63). Thus, soil fungi are generally more resistant to water stress than bacteria and can tolerate larger drought stresses (64). Also, soil microbial communities can respond differently to short- and long-term shifts in seasonal precipitation patterns. For instance, we recently demonstrated that soil microbes previously subjected to more than 40 years of water stress could better cope with subsequent water stresses (31, 65). Some reports have shown that soil bacterial communities are more strongly influenced by short-term temporal precipitation variability, while soil fungal communities are less responsive to short-term soil moisture pulses but have an increased abundance under long-term seasonal changes in precipitation patterns (66). Consequently, it appears that the effect of precipitation regimes on the diversity, structure, and composition of microbial communities depends on the initial community characteristics and the temporal extent of the stressful event.

In this study, we hypothesized that due to the potential differences in phylogenetic and physiological characteristics, different microbial taxa would respond differently to variations in water availability, leading to shifts in the diversity, structure, and composition of the microbial communities and ultimately affecting crop growth. We also hypothesized that the responses of microbial communities to variations in precipitation would differ between soils associated with drought-tolerant and drought-sensitive wheat cultivars. To test these hypotheses, we designed a field experiment where two wheat cultivars (drought-sensitive and -tolerant cultivars) were seeded, subjected to four different precipitation exclusion (PE) regimes (0%, 25%, 50%, and 75% exclusion), and sampled every 2 weeks from May to August. The objectives of this study were to determine how soil microbial communities respond to the manipulation of total precipitation versus the natural temporal dynamics of precipitation within a single growing season and if this differs between different wheat cultivars.

## RESULTS

### Precipitation and air temperature.

The mean biweekly precipitation was not significantly different among the sampling dates (2.5 mm, 2.4 mm, 3.7 mm, 4.4 mm, 0.8 mm, 4.6 mm, and 8.2 mm on 10 May, 24 May, 7 June, 21 June, 5 July, 19 July, and 1 August, respectively) (*P* = 0.783) despite a steep decrease on 5 July and an increase from 19 July to 1 August (see Fig. S1 in the supplemental material). In contrast, the mean biweekly temperature increased significantly (*P < *0.001) along the sampling dates (14.2°C, 15.9°C, 17.0°C, 20.2°C, 26.7°C, 24.0°C, and 23.4°C on 10 May, 24 May, 7 June, 21 June, 5 July, 19 July, and 1 August, respectively) (Fig. S1).

10.1128/msystems.00247-22.1 FIG S1 Variations in mean biweekly precipitation (A) and temperature (B) across different sampling dates within one growing season. Download FIG S1, DOCX file, 0.08 MB. Copyright © 2022 Wang et al. 2022 Wang et al. https://creativecommons.org/licenses/by/4.0/ This content is distributed under the terms of the Creative Commons Attribution 4.0 International license.

**FIG 1:**
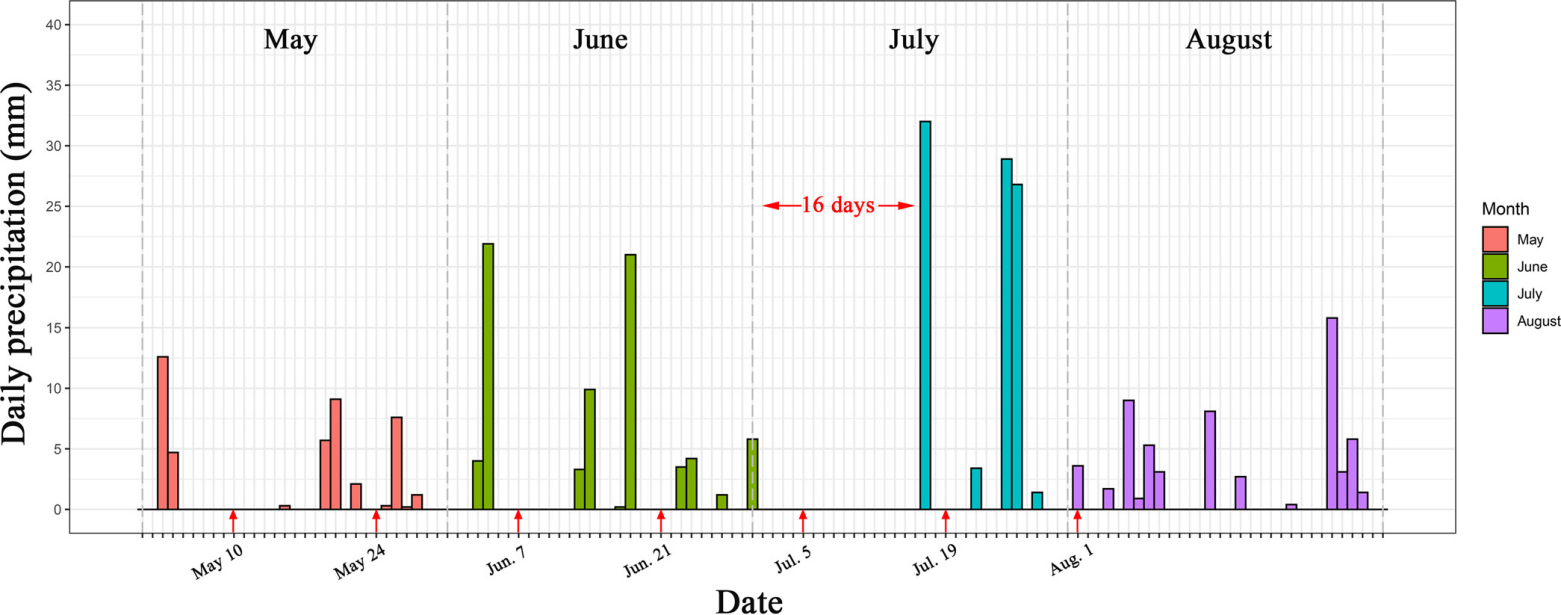
Variations in daily precipitation across the whole growing season (1 May to 31 August) in 2018 and the seven sampling dates, marked by red arrows. The daily precipitation data were retrieved from the weather station of the Montreal International Airport (QC), Canada (Station CWTQ, 45.4678, −73.7417 [http://www.agrometeo.org/indices/mcd/cwtq]), located 8.4 km away from our experimental field.

Variations in daily precipitation and the corresponding sampling dates across the whole growing season are shown in Fig. 1. The mean daily precipitations in May, June, July, and August were 1.4 mm, 2.5 mm, 3.0 mm, and 2.0 mm, respectively. The highest mean daily precipitation was in July, which was 2.1 times, 1.2 times, and 1.5 times higher than those in May, June, and August, respectively. Besides the highest mean daily precipitation in July, the precipitation in this month showed an extremely uneven distribution, with a 16-day dry spell followed by three >25-mm rain events in the second half of the month, which were the most significant rain events of the whole growing season (Fig. 1). In contrast, the precipitation distribution in May, June, and August was relatively well spread, with small and repeated rain events (Fig. 1).

The daily precipitations across the growing seasons of the last decade are shown in Fig. S2. The mean daily precipitation across the growing season fluctuated, ranging from 2.2 mm in 2012 to 3.9 mm in 2011. The highest monthly precipitation occurred 1 time in May, 4 times in June, 3 times in July, and 2 times in August (Fig. S2). Dry spells >10 days long occurred around 20 times across all years (2 times per year on average) (Fig. S2). In 2010, there were 3 dry spells, in May, July, and August (Fig. S2). Dramatic rewetting events following dry spells, like the one observed in 2018, occurred once per year on average at different moments of the growing season, from early June to mid-August (Fig. S2).

10.1128/msystems.00247-22.2 FIG S2 Variations in daily precipitation across the whole growing season (1 May to 31 August) in the past decade (2008 to 2017). Download FIG S2, DOCX file, 0.3 MB. Copyright © 2022 Wang et al. 2022 Wang et al. https://creativecommons.org/licenses/by/4.0/ This content is distributed under the terms of the Creative Commons Attribution 4.0 International license.

### Soil water content.

**FIG 2:**
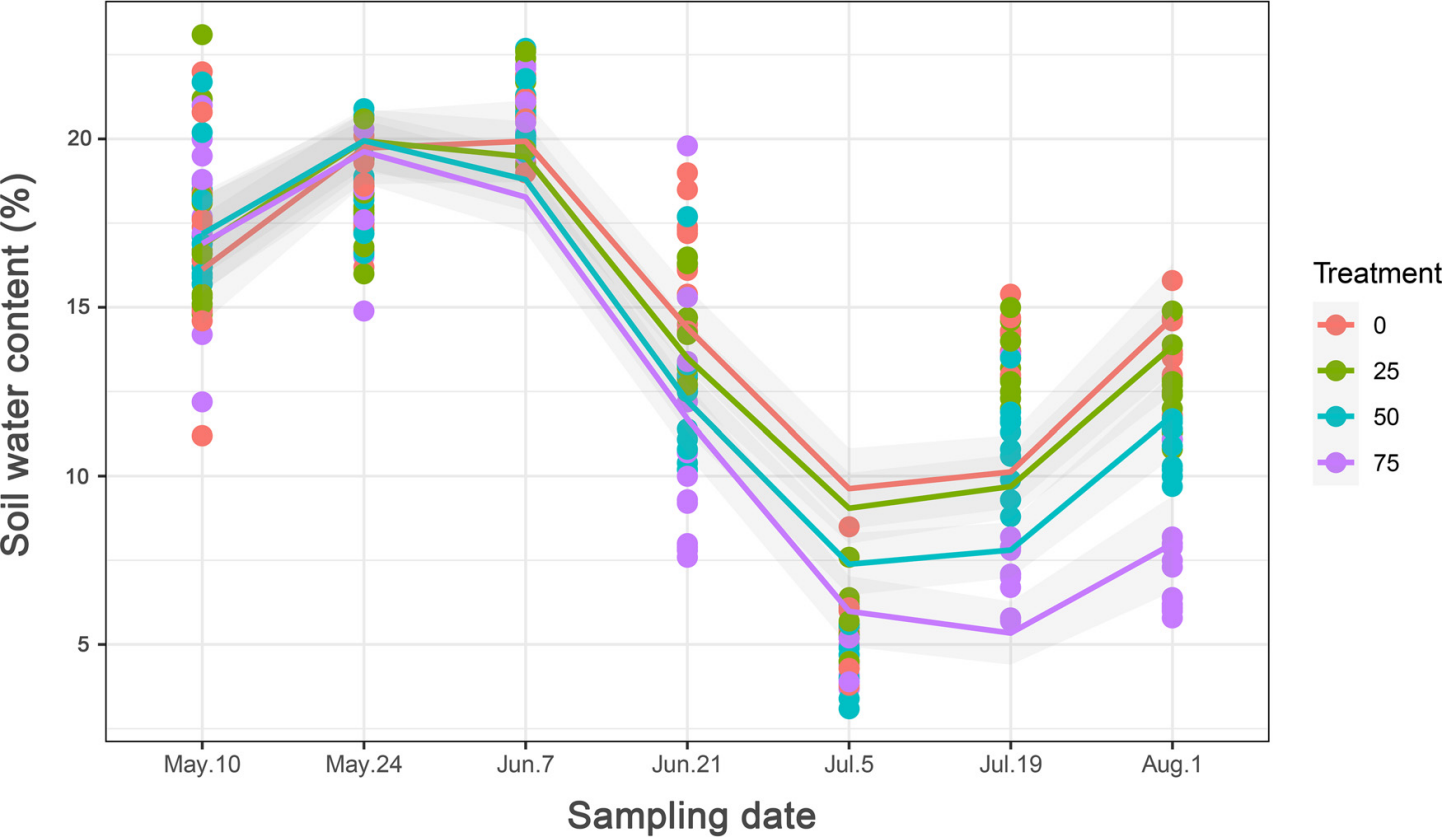
Variations in soil water content under each precipitation exclusion (PE) treatment across sampling dates in 2018. Points and fit curves are colored by PE treatment with 0%, 25%, 50%, and 75% exclusion.

The variations in soil water content mirrored the variations in mean biweekly precipitation (Fig. 2 and Fig. S2). Within each precipitation exclusion (PE) treatment, the soil water content differed significantly among sampling dates (*P < *0.001). The soil water content was relatively high for the 10 May, 24 May, and 7 June samplings compared to the other sampling dates but decreased steeply from 21 June to 5 July (Fig. 2). In accordance with the precipitation data, for all PE treatments, the soil water content was lowest for the 5 July sampling and then increased from 19 July to 1 August. The soil water content did not differ significantly among PE treatments for the 10 May, 24 May, and 7 June samplings (*P* = 0.839, 0.641, and 0.527, respectively), whereas it differed significantly for the 21 June (*P *< 0.001), 5 July (*P* = 0.014), and 1 August (*P* < 0.001) samplings.

### Wheat yields and grain protein content.

The grain protein content of the drought-sensitive wheat cultivar differed significantly among PE treatments (*P* = 0.028), with 50% PE treatments being significantly and marginally significantly higher than the 0% (*P* = 0.012) and 25% (*P* = 0.077) PE treatments (Fig. S3). In contrast, the grain protein content of the drought-tolerant wheat cultivar did not differ significantly among the PE treatments (*P* > 0.05), but the 25% PE treatment was marginally significantly higher than the 0% PE treatment (*P* = 0.078) (Fig. S3). No significant differences in wheat yields among the PE treatments were found for both the drought-sensitive and drought-tolerant cultivars (*P* > 0.05), whereas the yields of the drought-sensitive cultivar subjected to the 75% PE treatment were marginally significantly lower than those with the 25% (*P* = 0.092) and 50% (*P *= 0.056) PE treatments (Fig. S3).

10.1128/msystems.00247-22.3 FIG S3 Variations in yields and grain proteins of two wheat cultivars across precipitation exclusion treatments (0%, 25%, 50%, and 75% exclusion). Download FIG S3, DOCX file, 0.1 MB. Copyright © 2022 Wang et al. 2022 Wang et al. https://creativecommons.org/licenses/by/4.0/ This content is distributed under the terms of the Creative Commons Attribution 4.0 International license.

### Alpha diversity of the soil microbial communities.

**FIG 3:**
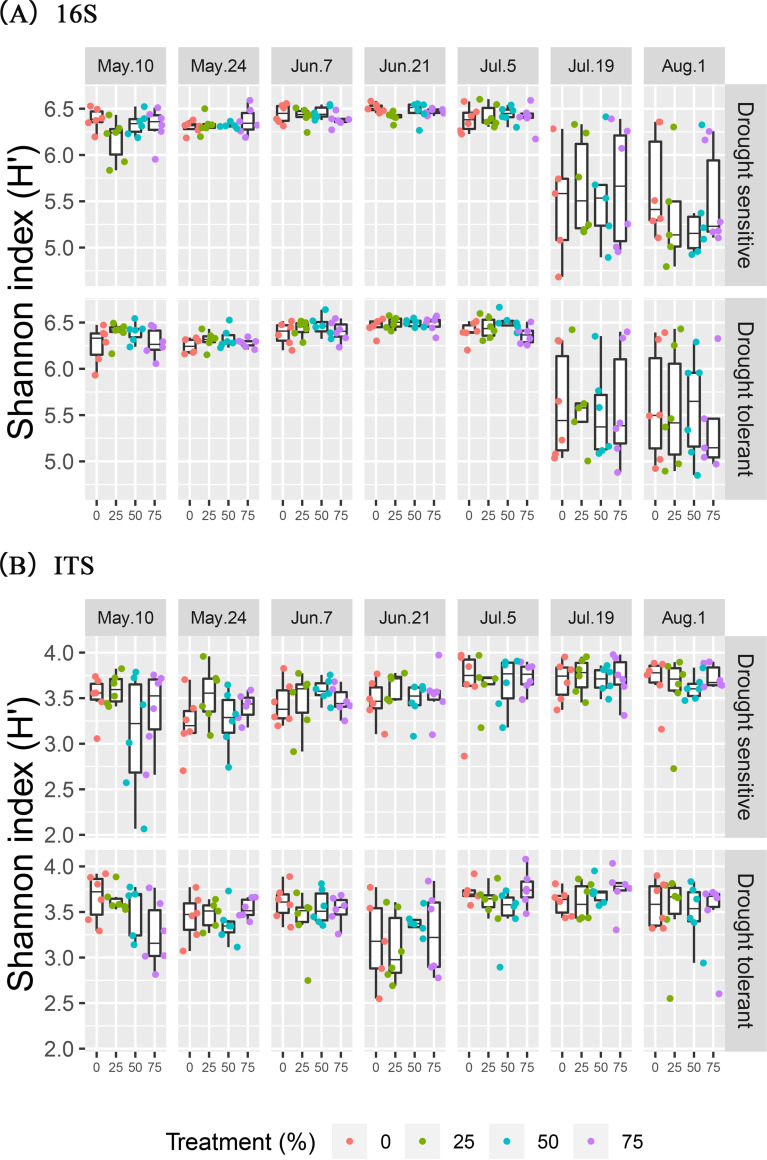
Shannon diversity of the bacterial and archaeal (16S rRNA gene amplicon sequencing) and fungal (ITS region amplicon sequencing) communities across precipitation exclusion (PE) treatments, sampling dates (10 May, 24 May, 7 June, 21 June, 5 July, 19 July, and 1 August), and wheat cultivars (drought-sensitive and -tolerant cultivars). Points represent the samples in each group colored by PE treatments with 0%, 25%, 50%, and 75% exclusion. The box plot shows quartile values.

The sequences obtained from the 16S rRNA gene and the internal transcribed spacer (ITS) region were grouped into 44,502 operational taxonomic units (OTUs) and 2,683 OTUs with a 97% sequence similarity threshold, respectively. Bacterial and archaeal as well as fungal alpha diversity estimated by the Shannon index, the inverse Simpson index, and phylogenetic diversity (PD) did not differ significantly between PE treatments or between cultivars (*P* > 0.05) (Table 1). In contrast, the Shannon (*P *< 0.001) and inverse Simpson (*P *< 0.001) diversity indices and Faith’s PD (*P* < 0.001) of bacterial, archaeal, and fungal communities varied significantly with sampling dates (Table 1). The interaction effect between PE treatments and sampling dates was marginally significant for the Shannon (*P* = 0.076) and significant for the Simpson (*P* = 0.036) diversity indices of fungal communities (Table 1), suggesting that the effect of the precipitation manipulation of fungal communities was dependent on the level of precipitation received and on the soil temperature and plant growth stage. The interaction effect between sampling date and wheat cultivar was significant for the Shannon (*P* = 0.006) and Simpson (*P* = 0.009) diversity indices of fungal communities (Table 1). In comparison to the small but significant variations observed in fungal diversity across sampling dates, bacterial and archaeal diversity significantly decreased on 19 July and 1 August (*P* < 0.001) (Fig. 3).

**TABLE 1:** F-ratios and significance levels from three-way repeated-measures ANOVA for bacterial and archaeal (16S rRNA gene amplicon sequencing) and fungal (ITS region amplicon sequencing) Shannon index, inverse Simpson index, and Faith’s PD^*a*^

Factor	16S			ITS		
	Shannon index	Inverse Simpson index	PD	Shannon index	Inverse Simpson index	PD
Treatment	0.11	0.02	1.93	0.07	0.01	0.18
Date	47.51***	40.41***	45.60***	6.59***	3.75**	4.09***
Cultivar	0.00	0.02	0.19	2.58	1.61	0.47
Treatment × date	0.52	0.46	0.51	1.93·	2.28*	1.03
Date × cultivar	0.38	0.31	0.50	3.05**	2.87**	0.91
Treatment × cultivar	0.05	0.01	1.13	0.18	0.16	0.14
Treatment × date × cultivar	0.22	0.06	0.59	0.33	0.42	0.07

a Treatment, treatments with precipitation exclusion (0%, 25%, 50%, and 75%); date, sampling dates (10 May, 24 May, 7 June, 21 June, 5 July, 19 July, and 1 August 2018); cultivar, drought-sensitive wheat and drought-tolerant wheat. ·, *P *< 0.1; *, *P *< 0.05; **, *P *< 0.01; ***, *P *< 0.001.

### Beta diversity and composition of the soil microbial communities.

**FIG 4:**
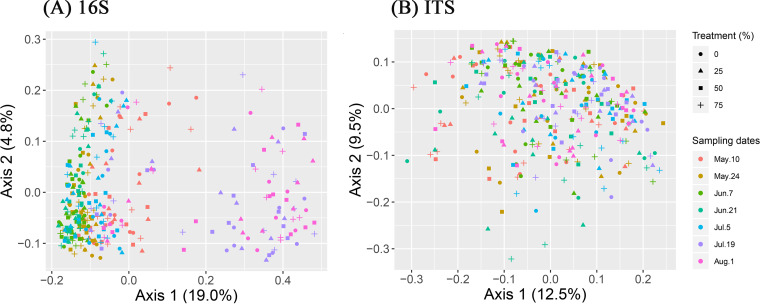
Principal-coordinate analysis (PCoA) of the bacterial and archaeal (16S rRNA gene amplicon sequencing) and fungal (ITS region amplicon sequencing) communities based on Bray-Curtis dissimilarity at the OTU level.

Bacterial, archaeal, and fungal community structures across PE treatments and sampling dates are represented on the first two axes of the principal-coordinate analysis (PCoA) plots based on the Bray-Curtis dissimilarity index (Fig. 4). For bacterial and archaeal PCoAs, the first axis clearly separates the 19 July and 1 August sampling dates from all of the other sampling dates (Fig. 4A). Despite unclear groupings in the fungal PCoA plots (Fig. 4B), the fungal community structure was significantly affected by PE treatments (*P* = 0.015), sampling dates (*P *= 0.0001), and wheat cultivars (*P* = 0.028), as shown by analysis of similarity (ANOSIM) (Table 2). In contrast, bacterial and archaeal community structure was not affected by PE treatments (*P *= 0.484) and wheat cultivars (*P *= 0.309) but was significantly different among sampling dates (*P* = 0.0001) (Table 2).

**TABLE 2:** ANOSIM of the bacterial and archaeal (16S rRNA gene amplicon sequencing) and fungal (ITS region amplicon sequencing) community structures based on Bray-Curtis dissimilarity across precipitation exclusion treatments, sampling dates, and cultivars^*a*^

Parameter	Value					
	16S			ITS		
	Treatments	Dates	Cultivars	Treatments	Dates	Cultivars
*R* value	−0.001	0.400	0.001	0.010	0.071	0.007
*P* value	0.484	0.0001	0.309	0.015	0.0001	0.028

a Treatments, treatments with precipitation exclusion (0%, 25%, 50%, and 75%); Dates, sampling dates (10 May, 24 May, 7 June, 21 June, 5 July, 19 July, and 1 August 2018); Cultivars, drought-sensitive wheat and drought-tolerant wheat.

**FIG 5:**
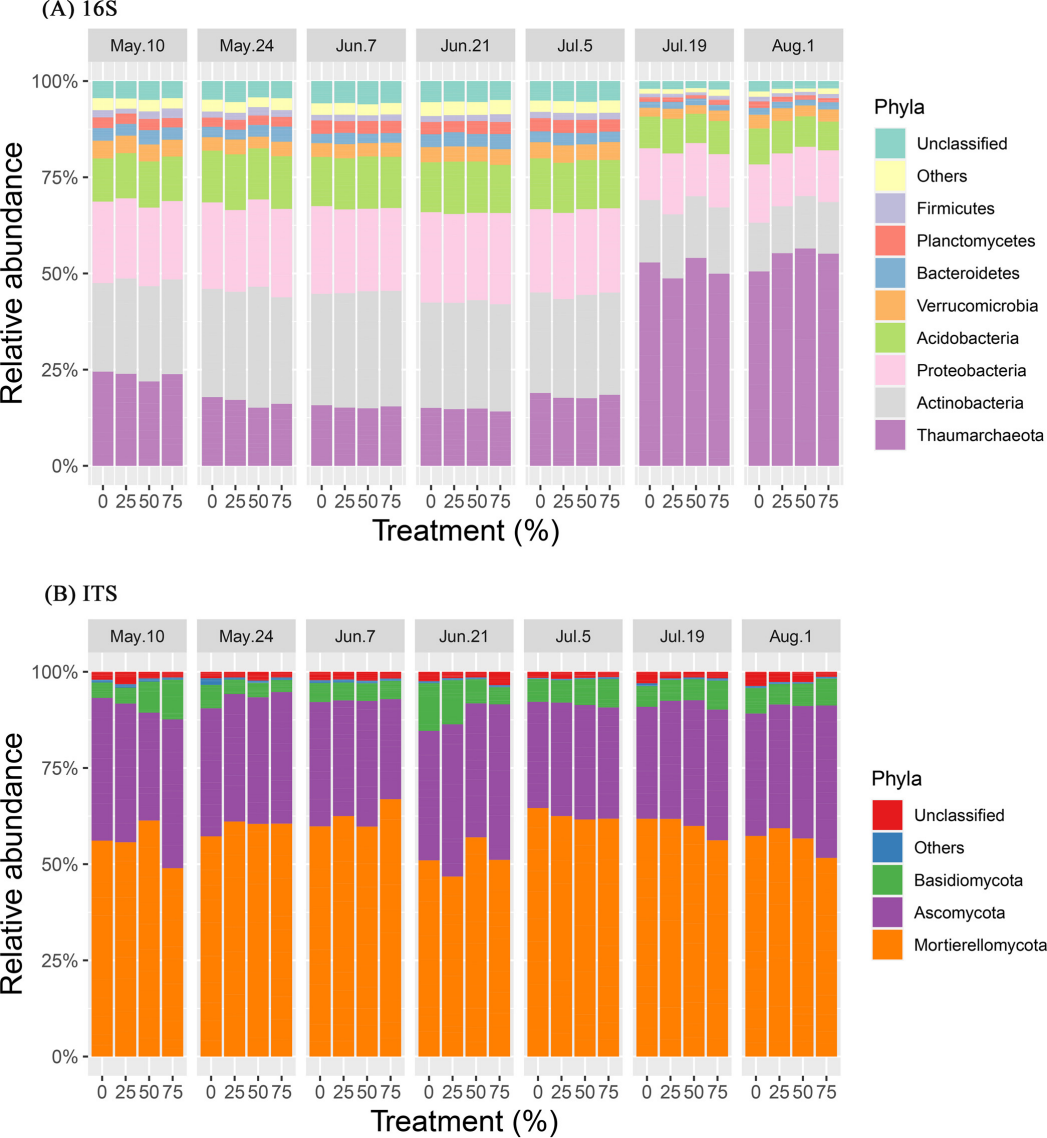
Bacterial and archaeal (A) and fungal (B) community compositions at the phylum level (mean relative abundance > 1%). The mean relative abundance was calculated based on Illumina amplicon sequencing of the 16S rRNA gene for bacteria and archaea and the ITS region for fungi.

At the phylum level, the bacterial and archaeal communities were dominated by members of the Thaumarchaeota, Actinobacteria, Proteobacteria, Acidobacteria, Verrucomicrobia, Bacteroidetes, Planctomycetes, and Firmicutes (Fig. 5A), whereas the fungal communities were dominated by the Mortierellomycota, Ascomycota, and Basidiomycota (Fig. 5B) (mean relative abundance of >1% across all samples). The relative abundances of all the dominant phyla in bacterial, archaeal, and fungal communities were not affected by PE treatments, whereas most were significantly different among sampling dates (*P* < 0.001) (Table 3). The relative abundance of Thaumarchaeota significantly increased on 19 July and 1 August (*P *< 0.001), whereas the relative abundance of other dominant bacterial phyla significantly decreased for these dates (*P *< 0.001) (Fig. 5A), probably explaining the patterns observed in alpha diversity and the PCoA. The interaction effect of PE treatments and wheat cultivars were significant for the Verrucomicrobia (*P *= 0.030), whereas the interaction effect of PE treatments and sampling dates was significant for the Basidiomycota (*P* = 0.004) (Table 3), suggesting some effect of the PE treatments for these two particular groups. The interaction effect between sampling dates and wheat cultivars was also significant for Bacteroidetes (*P* = 0.029) and Ascomycota (*P* = 0.020) (Table 3).

**TABLE 3:** F-ratios and significance levels from three-way repeated-measures ANOVA for the relative abundances of dominant phyla (>1%) of bacterial, archaeal, and fungal communities^*a*^

Phylum	Relative abundance						
	T	D	C	T × D	D × C	T × C	T × C × D
16S							
* Thaumarchaeota*	0.034	56.68***	0.008	0.246	0.361	0.051	0.263
*Actinobacteria*	2.039	32.59***	0.113	0.047	0.285	1.248	0.115
*Proteobacteria*	0.954	38.84***	0.106	0.562	0.270	0.643	0.581
*Acidobacteria*	1.043	23.55***	0.051	0.874	0.670	1.489	0.260
*Verrucomicrobia*	0.010	5.907***	1.812	0.483	0.297	4.739*	0.186
*Bacteroidetes*	2.124	7.969***	0.117	1.591	2.384*	1.521	1.242
*Planctomycetes*	2.142	28.64***	1.443	0.761	0.546	0.007	0.557
*Firmicutes*	2.534	3.518**	0.140	0.551	0.062	3.044·	1.083
ITS							
Mortierellomycota	0.620	5.924***	0.414	1.257	1.779	0.409	0.314
Ascomycota	1.597	4.708***	0.010	1.251	2.562*	1.049	0.280
Basidiomycota	0.020	0.822	1.242	3.291**	0.344	0.032	0.312

a T, treatments with precipitation exclusion (0%, 25%, 50%, and 75%); D, sampling dates (10 May, 24 May, 7 June, 21 June, 5 July, 19 July, and 1 August 2018); C, drought-sensitive wheat cultivar and drought-tolerant wheat cultivar. ·, *P < *0.1; *, *P < *0.05; **, *P < *0.01; ***, *P < *0.001 (significance determined by ANOVA).

At finer taxonomical levels, the relative abundances of the dominant bacterial, archaeal, and fungal genera (mean relative abundance of >1% across all samples) also varied significantly across PE treatments, sampling dates, and wheat cultivars (Table 4). The effect of PE treatments was significant only for the verrucomicrobial genus *Terrimicrobium* (*P* = 0.024) and marginally significant for the acidobacterial genus Gp6 (*P* = 0.094) and the basidiomycotal genus *Ganoderma* (*P* = 0.085) (Table 4). The effect of sampling dates was significant or marginally significant for all 10 bacterial and archaeal genera but was significant for only the fungal genera *Mortierella* (*P *< 0.001), *Gliomastix* (*P* = 0.002), and *Ganoderma* (*P* = 0.003) (Table 4). The interaction effect between PE treatments and wheat cultivars was significant for the bacterial genera *Solirubrobacter* (*P* = 0.041), *Hyphomicrobium* (*P* = 0.016), and *Terrimicrobium* (*P* = 0.024) and the fungal genus *Thelonectria* (*P* = 0.044) and marginally significant for the bacterial genus *Arthrobacter* (*P* = 0.059) (Table 4). The three-way interaction was significant only for the fungal genus *Neosetophoma* (*P* = 0.020) (Table 4).

**TABLE 4:** F-ratios and significance levels from three-way repeated-measures ANOVA of the relative abundances of dominant genera (>1%) of bacterial, archaeal, and fungal communities^*a*^

Genus	Relative abundance						
	T	D	C	T × D	D × C	T × C	T × C × D
16S							
*Nitrososphaera*	0.034	56.68***	0.008	0.246	0.361	0.051	0.263
*Gaiella*	2.715	22.75***	0.934	0.557	0.545	1.119	0.608
Gp6	2.828·	14.06***	0.014	0.548	0.711	0.226	0.241
Gp16	2.681	23.09***	0.000	1.114	0.654	1.840	0.122
*Solirubrobacter*	1.804	8.867***	0.318	0.237	0.055	4.207*	0.283
*Arthrobacter*	0.959	2.566*	3.365·	1.164	0.359	3.604·	0.804
*Spartobacteria*	0.886	2.034·	2.762·	0.225	0.208	0.188	0.245
*Hyphomicrobium*	2.424	5.656***	0.085	0.756	1.233	5.921*	0.452
*Conexibacter*	1.477	12.01***	0.032	1.104	1.067	0.246	0.789
*Terrimicrobium*	5.178*	2.087·	0.293	0.470	0.575	5.166*	1.126
ITS							
*Mortierella*	0.645	5.872***	0.449	1.297	1.801	0.530	0.343
*Gliomastix*	0.010	3.623**	0.948	0.177	0.275	0.780	0.233
*Ganoderma*	2.995·	3.411**	4.140*	1.940·	0.361	0.199	0.506
*Pezizella*	2.197	1.711	0.272	0.925	0.959	0.596	1.445
*Neosetophoma*	0.113	0.875	1.867	1.714	0.131	0.513	2.543*
*Thelonectria*	0.272	0.444	0.069	0.831	1.579	4.080*	0.685

a T, treatments with precipitation exclusion (0%, 25%, 50%, and 75%); D, sampling dates (10 May, 24 May, 7 June, 21 June, 5 July, 19 July, and 1 August 2018); C, drought-sensitive wheat cultivar and drought-tolerant wheat cultivar. ·, *P < *0.1; *, *P < *0.05; **, *P < *0.01; ***, *P < *0.001 (significance determined by ANOVA).

**FIG 6:**
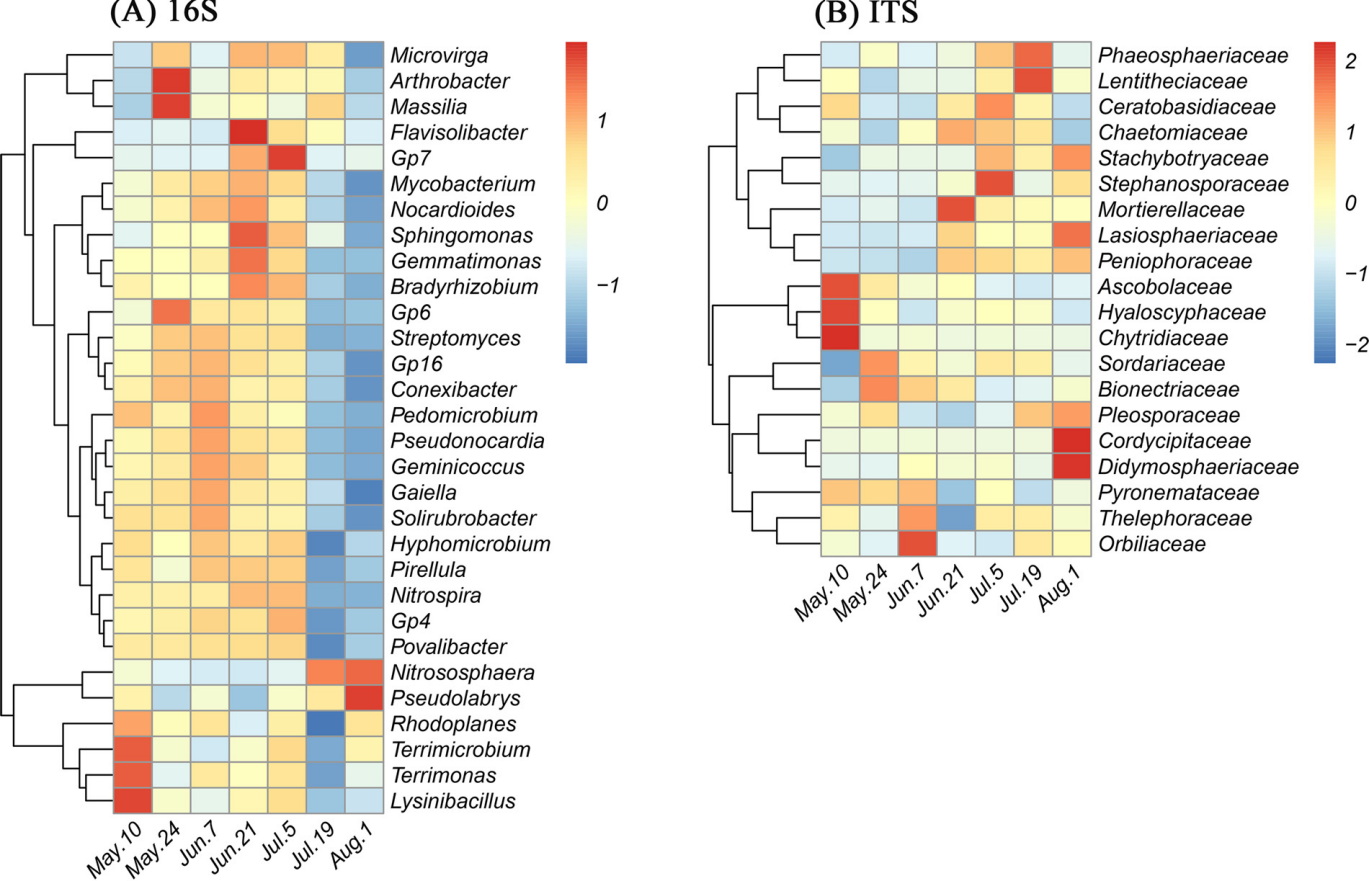
Heatmaps showing the abundance distributions of the top 30 archaeal and bacterial genera and 20 fungal families based on 16S rRNA gene (A) and ITS region (B) amplicon sequencing across seven sampling dates (10 May, 24 May, 7 June, 21 June, 5 July, 19 July, and 1 August). Heatmaps are color-coded based on row *z*-scores with default clustering methods (Euclidean distances).

As most dominant microbial taxa were significantly affected by the sampling dates, a heatmap representation was created to illustrate the variations in the relative abundances of dominant members of the bacterial, archaeal, and fungal communities along the seven sampling dates. Overall, all 30 bacterial and archaeal genera showed significant differences in their relative abundances across sampling dates (*P *< 0.05) (Fig. 6A). The relative abundances of most bacterial and archaeal genera on 19 July and 1 August were considerably lower than those on the other sampling dates (*P* < 0.05), but *Nitrososphaera* showed the inverse trend, with exceptionally high relative abundances on both 19 July and 1 August (*P* < 0.001) (Fig. 6A). In contrast, the relative abundances of *Terrimicrobium*, *Terrimonas*, and *Lysinibacillus* on 10 May and *Arthrobacter* and *Massilia* on 24 May were significantly higher than those on the other sampling dates (*P* < 0.05) (Fig. 6A). Like the bacterial and archaeal genera, the relative abundances of all 20 fungal families were also significantly different between sampling dates (*P* < 0.05), but the variations in the relative abundances of each taxonomic group were distinctly different along sampling dates (Fig. 6B).

## DISCUSSION

It is known that microbe-mediated soil processes are driven largely by precipitation/water availability. The potential impact of spatial and intra-annual temporal variations in precipitation on microbial communities has been extensively examined during the past decades (66–68). In this study, however, we focused mainly on the shifts in soil microbial communities under experimentally manipulated precipitation regimes during a single growing season with a relatively high-resolution sampling scheme, which was rarely addressed to date. Our study highlights the uneven responses of soil fungal, bacterial, and archaeal communities and their associated taxa to experimental manipulation of the precipitation regime, within an overriding effect of time.

Even though it significantly changed the soil water content on some sampling dates (e.g., 21 June, 5 July, and 1 August) and some microbial parameters, the short-term effects of precipitation manipulation on microbial communities were dwarfed by the effects of sampling date. Sampling date in the context of our experiment is probably related to three main factors: (i) precipitation/soil water content, (ii) plant growth stage, and (iii) soil temperature. Based on local meteorological records, precipitation was relatively well spread in May and June, leading to a relatively steady soil water content of around 10 to 20%. There was then a 16-day dry spell from 1 to 16 July, resulting in a sharp decrease in the soil water content down to approximately 5%, followed by a 2-fold increase in soil moisture from mid-July to early August (back to an average of 11% on 1 August). This short-term drying-rewetting event is most probably behind the patterns observed in soil microbial communities, as these types of events were shown to cause a nutrient pulse followed by a sharp increase in soil respiration and mineralization (29, 34). Moreover, increasing plant biomass, the consequent increases in belowground C allocation, as well as the decomposition of plant litter and soil organic matter (69–71) can also stimulate microbial growth and activities through increased depletion of C substrates (72). Plant growth stage has also been shown to influence rhizosphere microbial communities (73, 74). Even though we did not sample the rhizosphere *per se*, we did observe some cultivar effects, suggesting that the plant influence extends past the narrowly defined rhizosphere (75), especially for densely planted crops. Plants also respond to weather events by modulating their rhizodeposition (76, 77), which could have resulted in an indirect effect of weather patterns on microbial communities. These plant-mediated effects could thus explain part of the strong temporal patterns observed in microbial communities. In addition, soil temperature can vary with season and soil water conditions, which would accordingly affect soil microbial activity and respiration. A large number of warming or multifactor global change experiments have demonstrated that precipitation/soil water availability could suppress or stimulate microbial respiration, litter decomposition, plant biomass, and root activity via direct or indirect influences on soil temperature (78–80). Although we did not monitor the daily changes in soil temperature, the mean biweekly temperature retrieved from meteorological data did show a step-by-step increase from May to August, suggesting potentially increased evapotranspiration regardless of what precipitation regimes we used. The variation in soil temperature might thus be another explanation for the observed effects of sampling date. Taken together, temporal shifts in precipitation and soil moisture, plant-microbe interactions, and soil temperature probably explain the overriding effect of time on the diversity and composition of microbial communities.

We had hypothesized that various taxa would respond differently to variations in precipitation owing to their physiological differences, which would result in changes in microbial community diversity, structure, and composition. Our findings have provided strong evidence to support this hypothesis. First, although both bacterial and archaeal diversity and fungal diversity were not significantly affected by precipitation manipulations, they did not show similar shifts across sampling dates. A dramatic decrease in bacterial and archaeal diversity was found on 19 July and 1 August, which, as mentioned above, followed a strong rewetting event. These events were shown to reduce bacterial and archaeal diversity by favoring the portion of the community that is better adapted to cope with this type of stress (59). Unlike bacterial and archaeal diversity, fungal diversity did not change across sampling times, even though the fungi also experienced the same rewetting event. This finding is consistent with previous reports that showed that soil fungi were generally more resistant to drought or short-term dry-wet alternations than bacteria because of their different physiological strategies for coping with these stresses (14, 34). The differences between the responses of the bacterial and archaeal communities’ and the fungal community’s structure and composition to precipitation manipulation and time provide further evidence supporting this point. In accordance with the variation in diversity, the bacterial and archaeal communities were clearly different between the 19 July and 1 August samplings and all other samplings, suggesting that the bacterial and archaeal community responses to time resulted in not only reduced diversity but also a shift in community assembly, which would shape a community that can withstand new environmental conditions or stresses (52). In contrast, the fungal community did not exhibit such a clear alteration in response to time, although sampling dates as well as precipitation manipulation were both found to significantly affect fungal community structure by ANOSIM. This is in agreement with previous studies that suggested that soil fungi could have a better “buffer” capability for coping with water stresses than soil bacteria or archaea due to physiological differences in water acquisition mechanisms (64).

In addition to microbial community diversity and structure, taxonomic composition was also strongly driven by time, with clear differences between bacteria and archaea and fungi. For the bacterial and archaeal communities, the relative abundances of most of the dominant phyla and genera were significantly affected by sampling dates and generally showed a reduced relative abundance in the 19 July and 1 August samples compared with all other samples. However, the relative abundance of the *Nitrososphaera* genus within the *Thaumarchaeota* phylum, a genus of ammonia-oxidizing archaea (AOA), was exceptionally high on those two sampling dates. Together with the dramatic reduction of bacterial and archaeal diversity in the 19 July and 1 August samples, these findings suggest that AOA became increasingly predominant, resulting in decreases in the relative abundances of most other dominant groups and the inability to detect certain rare species. Alternatively, the AOA could have simply maintained their population size while the rest of the community died or decreased in size. Yet this remarkable rise in the AOA relative abundance, probably due to the sharp rewetting of dry soils that occurred in late July, as mentioned above, could result in altered soil processes (e.g., increased nitrification rates), which would have to be confirmed by measuring actual activities. Previous studies have found that nitrifiers in soils are able to survive dry periods (39) and are highly sensitive to moisture stress (81), being able to increase their biomass and activity with the flush of NH_4_^+^ released during the rewetting of dry soil (82). This is probably an explanation for the increased relative abundance of the AOA community observed in our study, but the physiological adaptation of *Nitrososphaera* to moisture stress and drying-rewetting events warrants more studies for this observation to be conclusive. Alternatively, reduced competition for N caused by a reduction in wheat N requirements at the ripening stage, toward the end of the growing season, and a reduced microbial community could have left more N for surviving microbes, explaining the sudden increase in AOA. Whatever the underlying mechanism is, this increase in AOA relative abundance could have important consequences for wheat yields and grain quality. Indeed, in a recent study, we found significant negative correlations between AOA abundance early in the growing season and yield and grain baking quality (83). We had hypothesized that because of the lower mobility and passive absorbance by the plant and because it could be directly used for amino acid synthesis, ammonium was energetically preferable for higher grain quality. In the case of the current study, since the increase in AOA occurred late in the growing season, when wheat N requirements are less important, it would probably have fewer consequences than such an increase in a key growth stage with high wheat N requirements.

Likewise, the relative abundances of some bacterial genera such as *Arthrobacter*, *Massilia*, *Terrimicrobium*, *Terrimonas*, and *Lysinibacillus* were relatively high in early-spring samples (e.g., 10 May and 24 May), when air and soil temperatures were low and plants were absent or had little influence on soil microbes. The higher relative abundance of some of these genera could be related to stronger resistance to nutrient-deficient conditions. For example, numerous studies have demonstrated that the genus *Arthrobacter* has a high level of resistance to starvation (84) as well as the capability of using a variety of C substrates (85). For the fungal community, regardless of the strong effect of sampling dates on the relative abundances of certain taxa, we found no regular pattern in composition across different sampling times. This observation is coherent with the above-mentioned point that soil fungi are likely to be more stable than bacteria and archaea to short-term water variability. It should also be mentioned that since DNA-based methods are unable to distinguish between active and inactive or dead microbes, we probably detected many inactive microbes as the soil dried during the summer and, possibly, many dead microbes as the soil rewetted, which could have affected the patterns observed.

In agreement with the effects of precipitation regimes on microbial communities shown in some studies, precipitation manipulation in our study indeed affected the fungal community structure and the relative abundances of some microbial taxa, often in interactions with other experimental factors. The overall changes in soil water content due to precipitation manipulation were relatively small (at most 6.3% and at most sampling dates <1%) compared to the variation across the growing season (from an average of 5.2% on 5 July to an average of 20.4% on 7 June), which probably precluded the observation of more significant trends. It therefore appears that changes of a few percent in the soil water content do not seem to have large consequences for soil microbial communities as long as water is still available. However, our data suggest that drying-rewetting cycles could have much more important effects on soil microbial communities, most especially the AOA. This is in line with previous studies that compared the effects of temperature and freeze-thaw cycles and found that the latter had much greater consequences for the microbial communities and functions than the former (86). Thus, the additional variability in the climate caused by global changes will probably have more important consequences for soil microbial communities and, consequently, crop productivity and quality than small changes in soil moisture content. In our area of study (southern Québec), dry spells of 10 days or more occurred around 20 times during the growing seasons of the past decades (2 times per year on average). Dramatic rewetting events on dry soils like the one observed here occur 1 time per year on average anytime during the whole growing season, from early June to mid-August. If this were to increase, with, for instance, less precipitation in the spring, it could significantly affect soil microbial communities and, potentially, nutrient cycling in a moment where plant nutrient needs are important.

In conclusion, our findings provide evidence that soil microbial communities respond much more significantly to drying-rewetting cycles than small variations in precipitation/soil water content. Indeed, we observed a complete overhaul of the bacterial and archaeal communities following soil rewetting, including a dramatic increase in the relative abundance of the AOA genus *Nitrososphaera*. No such shifts were observed to be due to our precipitation manipulations. Furthermore, fungal communities showed a smaller response than bacteria and archaea, highlighting key differences between these two microbial groups, probably due to life strategies and major physiological differences. Our study implies that frequent large rewetting of dry soils will cause more rapid responses of soil microbial community structure and composition than regular decreased precipitation under erratic climate change pressures and, thus, will probably trigger dramatic changes in microbe-mediated soil nutrient cycles and ecosystem functioning.

## MATERIALS AND METHODS

### Field experiment and sampling.

**FIG 7:**
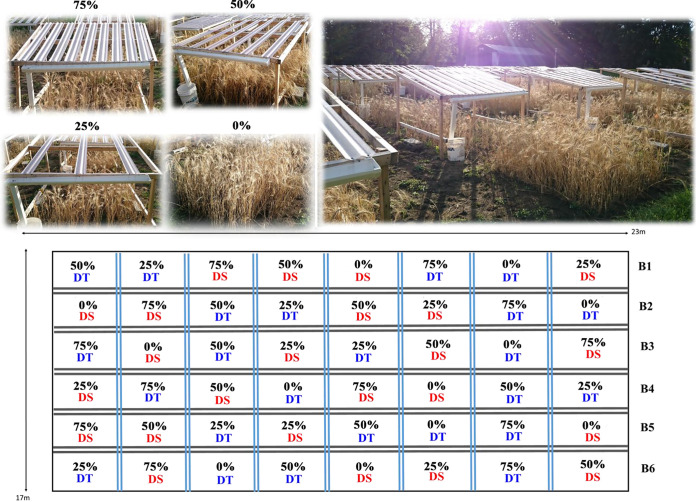
Field experiment of precipitation manipulation with four rainfall exclusion regimes (0%, 25%, 50%, and 75%) and two wheat cultivars (drought-sensitive [DS] and drought-tolerant [DT] cultivars) arranged in six blocks. The field experiment was set up at the Armand-Frappier Santé Biotechnologie Research Center (Québec, Canada) in 2016. A total of 48 plots (2 m by 2 m) were established based on a randomized complete block design.

The field trial was set up at the Armand-Frappier Santé Biotechnologie Research Center (Laval, QC, Canada) in 2016. Average daily rainfalls at this location between 1 May and 31 August were 2.5 mm, 3.5 mm, and 2.2 mm from 2016 to 2018, respectively. A total of 48 plots (2 m by 2 m) were established based on a randomized complete block design with four different rainfall exclusion regimes (0%, 25%, 50%, and 75% exclusion) and two wheat cultivars (drought-sensitive Triticum aestivum cv. AC Nass and drought-tolerant Triticum aestivum cv. AC Barrie) arranged in six blocks (Fig. 7). The rainfall exclusion treatments were performed using rainout shelters (Fig. 7), which were covered with various amounts of transparent plastic sheeting. The rain was intercepted by the plastic sheeting, guided into a gutter and downspout, and collected in 20-L buckets that were manually emptied following significant rainfall events. Soil sampling was carried out every 2 weeks on 10 May (at seedling time [time zero]), 24 May, 7 June, 21 June, 5 July, 19 July, and 1 August 2018. Soil samples were collected with five soil cores (2-cm diameter by 10-cm depth) of the upper 10-cm layer in each plot (4 treatments × 6 blocks × 2 cultivars × 7 sampling dates = 336 composite samples). Composite soil samples were sieved through a 2.0-mm sieve, placed into a sterile plastic bag, and immediately stored at −80°C in the laboratory prior to DNA extraction.

We measured soil water content by weighing soils before and after drying overnight at 105°C. Yields were measured from each plot by manually harvesting and threshing the grains and weighing them. The grain protein content was analyzed in the quality control laboratory of Les Moulins de Soulanges (St-Polycarpe, QC, Canada). Based on our experimental design and sampling strategy as well as the dimension of the experimental area, we did not measure any other soil physicochemical parameters such as pH, total carbon, nitrogen, and phosphorus, etc., because these environmental variables were not expected to vary substantially across the spatiotemporal scale of our experiment. To assess the variation in precipitation and temperature across a growing season (1 May to 31 August), we retrieved the mean biweekly precipitation and temperature in 2018 and the daily precipitation in 2018 and in the past decade (2008 to 2017) recorded by the weather station of the Montreal International Airport (QC), Canada (Station CWTQ, 45.4678, −73.7417 [http://www.agrometeo.org/indices/mcd/cwtq]), located 8.42 km away from our experimental field. For each sampling date, we retrieved the mean precipitation and temperature for the previous two weeks.

### DNA extraction and high-throughput amplicon sequencing.

Genomic DNA was extracted from 0.5 g of well-mixed soil for each sample using the DNeasy PowerLyzer PowerSoil kit (Qiagen) according to the manufacturer’s instructions. The quality of the extracted DNA was assessed based on 260/280-nm and 260/230-nm absorbance ratios obtained using a NanoDrop ND-1000 spectrophotometer (NanoDrop Technologies Inc., Thermo Scientific, USA). DNA was stored at −20°C until use for PCR.

PCR amplicon libraries were prepared for the bacterial and archaeal 16S rRNA genes using primers 515F and 806R targeting the V4 region (87) and for the fungal ITS1 region using primers ITS1F and 58A2R (88). The first step of PCR was performed using template-specific primers with a short adaptor sequence, and the second step of PCR was conducted with primers containing Illumina barcodes. Two PCR amplification steps were performed in a T100 thermal cycler (Bio-Rad, USA). Reagents and reaction conditions for the two PCR amplifications are shown in Table S1 in the supplemental material. Amplicons were visualized on a 1% agarose gel and purified using AMPure XP beads (Beckman Coulter, Indianapolis, IN, USA) according to the manufacturer’s instructions. PCR products from different samples were pooled into a composite library and sequenced on an Illumina MiSeq sequencer (2 × 250 pair-end reads) at the Centre d’Expertise et de Services Genome Québec (Montréal, Canada). Totals of 17,084,986 16S rRNA gene reads and 22,411,001 ITS region reads were produced.

10.1128/msystems.00247-22.4 TABLE S1 Reagents and reaction conditions for two PCR amplification steps. Download Table S1, DOCX file, 0.02 MB. Copyright © 2022 Wang et al. 2022 Wang et al. https://creativecommons.org/licenses/by/4.0/ This content is distributed under the terms of the Creative Commons Attribution 4.0 International license.

### Bioinformatics analysis.

Primers were trimmed with up to one mismatch allowed and starting position ≤1. Forward and reverse reads of the same sequence were merged with at least a 30-bp overlap and <0.25 mismatches by using FLASH v1.2.5 (89). The sequences were then quality trimmed using Btrim (90) with a Phred-score threshold of 30 over a 5-bp window size. Merged sequences with an ambiguous base or <240 bp for 16S and <200 bp for the ITS were discarded. Chimera sequences were detected and removed with the UCHIME algorithm (91). Operational taxonomic units (OTUs) were clustered by UPARSE at 97% identity (92) for both the 16S rRNA gene and ITS region. Representative sequences of OTUs were annotated taxonomically by the RDP 16S rRNA reference database (93) and the UNITE ITS reference database (94). All samples were rarefied to 2,052 and 2,030 sequences for the 16S rRNA gene and ITS using the number of reads of the sample with the smallest amounts of reads, respectively. The above-mentioned steps were performed using an in-house pipeline that was built on the Galaxy platform at the Research Center for Eco-Environmental Sciences, Chinese Academy of Sciences (http://mem.rcees.ac.cn:8080/root/) (95).

### Statistical analysis.

All statistical analyses and figure generation were performed in R (v.4.0.3). Faith’s phylogenetic diversity (PD) (96) was estimated using the pd function of the picante package (97). Shannon and inverse Simpson diversity indices were calculated with the alphaDiversity function of the otuSummary package. Pairwise Bray-Curtis dissimilarities between samples were calculated with the vegdist function of the vegan package (98). Principal-coordinate analysis (PCoA) was used to visualize similarity between samples based on bacterial and fungal composition using the cmdscale function of the vegan package. The dominant groups of microbial communities were selected based on the relative abundance assessment of the OTU table at the corresponding taxonomic level by the tax.abund function. After the removal of unclassified genera or families, a heatmap was generated using the pheatmap function to display the variation in the relative abundances of the top 30 genera for bacterial and archaeal communities and the top 20 families for fungal communities. The values of the cluster heatmap were scaled in the row direction using the Euclidean distance as a dissimilarity measure. The effects of the PE treatments, sampling dates, and wheat cultivars on the bacterial and fungal community structures were tested using the anosim function. Best-of-fit modeling of the regression was performed using the lm function. The bar charts, box plots, and scatterplots were generated using the ggplot, geom_boxplot, geom_bar, geom_point, and geom_smooth functions of the ggplot2 package.

Normal distributions of the residuals of the models were checked with the Shapiro-Wilk test using the Shapiro.test function. Depending on the distribution of the estimated parameters, the data were log or square-root transformed if they did not satisfy this assumption. Three-way repeated-measures analysis of variance (ANOVA) by the aov function or the Kruskal-Wallis rank sum test by the krusk.test function was used to check for significant differences in the diversity and relative abundances of dominant groups of microbial communities. One-way ANOVA with *post hoc* tests was also used to test the significant differences in mean biweekly precipitation and temperature among sampling dates using 14 daily values before each sampling date, soil moisture contents on each sampling date caused by precipitation manipulation, and grain protein contents and yields of wheat among PE treatments with the aov function.

### Data availability.

The raw sequences of the amplicon sequencing data were deposited in the NCBI Sequence Read Archive (SRA) database (www.ncbi.nlm.nih.gov/sra) under BioProject accession number PRJNA686206. The R code and related data files for all analyses are freely available as an archived GitHub repository at https://github.com/WangXB1999/repository.
