# Supplementary figures and images for: A Drying-Rewetting Cycle Imposes More Important Shifts on Soil Microbial Communities than Does Reduced Precipitation
Source: mSystems. 2022 Jun 28;7(4):e00247-22. doi: 10.1128/msystems.00247-22 (PMC9426475; doi:10.1128/msystems.00247-22)

**Figure S1**.


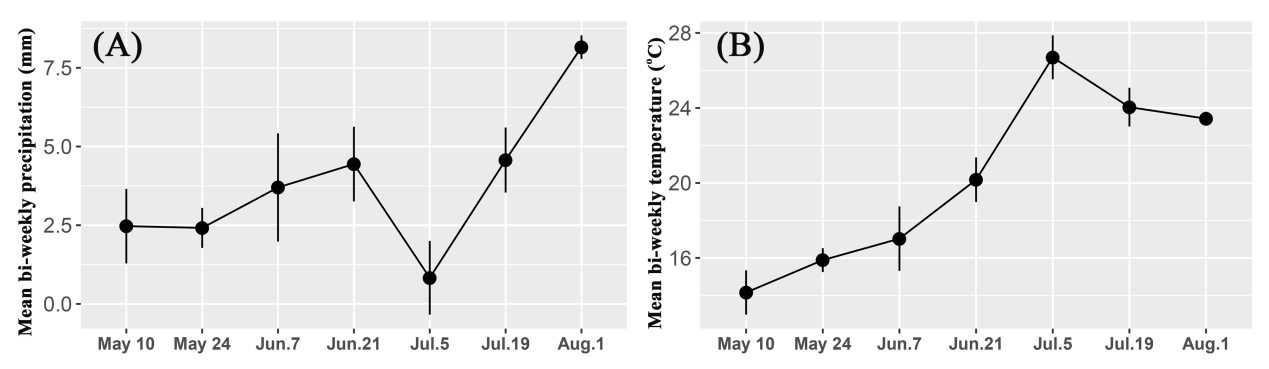

Supplement: FIG S1 [file msystems.00247-22-s0001.docx]
