# Supplementary material for: A Drying-Rewetting Cycle Imposes More Important Shifts on Soil Microbial Communities than Does Reduced Precipitation
Source: mSystems. 2022 Jun 28;7(4):e00247-22. doi: 10.1128/msystems.00247-22 (PMC9426475; doi:10.1128/msystems.00247-22)

**Figure S2**. Variations in daily precipitation across the whole growing season (May 1^st^ to August 31^st^) in the past decades (2008-2017).


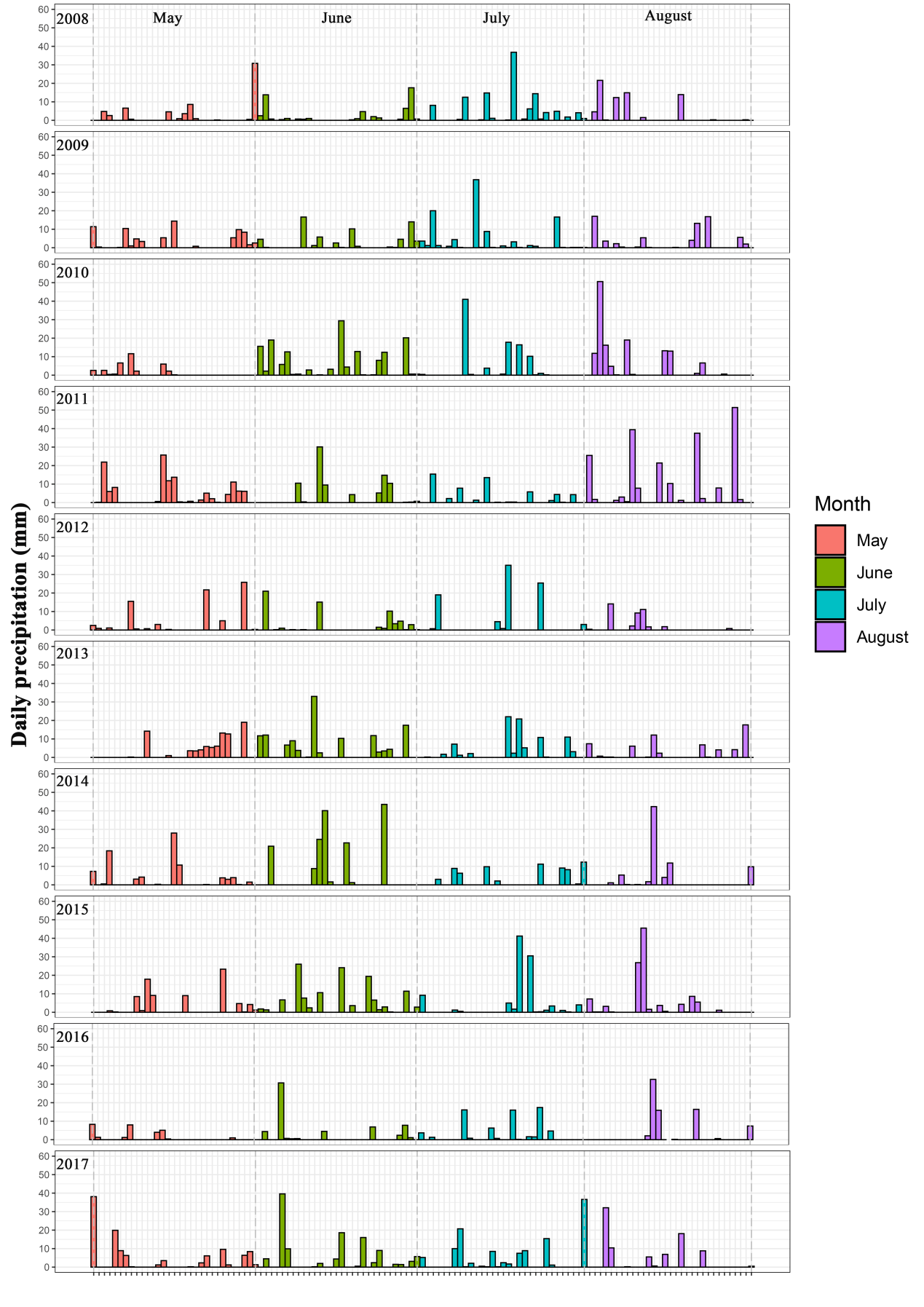

Supplement: FIG S2 [file msystems.00247-22-s0002.docx]
