# Supplementary material for: A Drying-Rewetting Cycle Imposes More Important Shifts on Soil Microbial Communities than Does Reduced Precipitation
Source: mSystems. 2022 Jun 28;7(4):e00247-22. doi: 10.1128/msystems.00247-22 (PMC9426475; doi:10.1128/msystems.00247-22)

**Figure S3**. Variations in yields and grain protein of two wheat cultivars across precipitation exclusion treatments (0%, 25%, 50% and 75% exclusion).


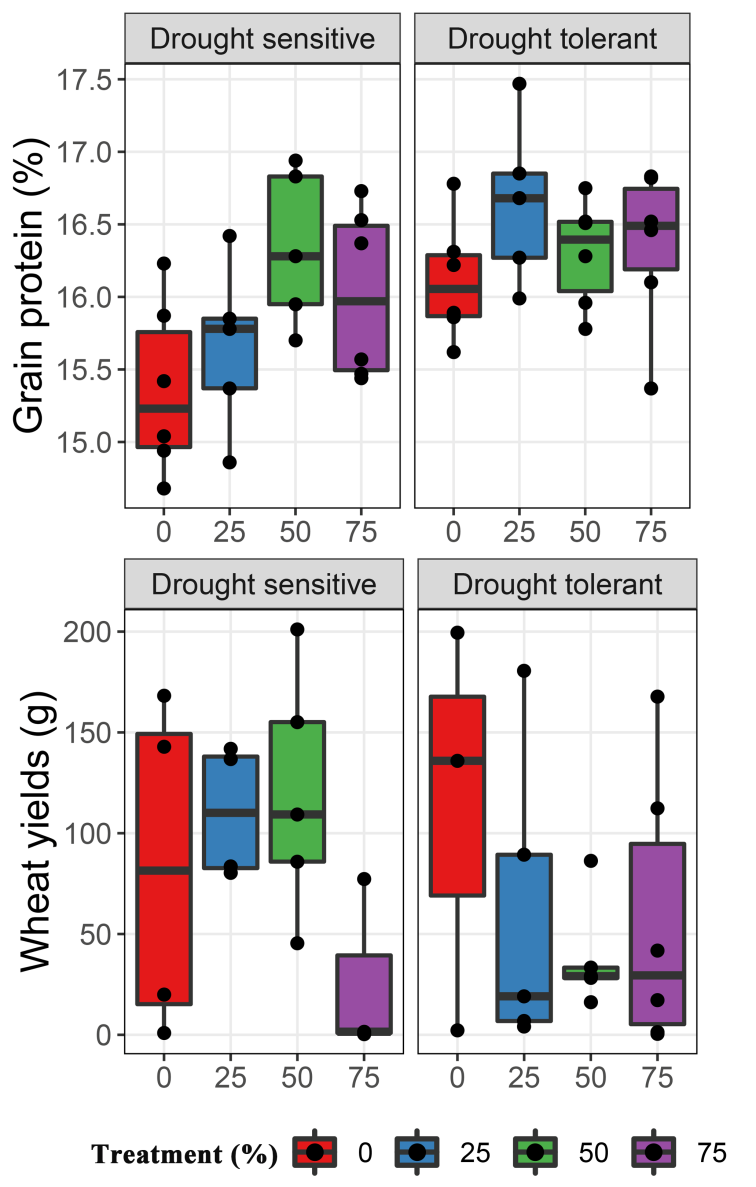

Supplement: FIG S3 [file msystems.00247-22-s0003.docx]
