# Supplementary material for: A Drying-Rewetting Cycle Imposes More Important Shifts on Soil Microbial Communities than Does Reduced Precipitation
Source: mSystems. 2022 Jun 28;7(4):e00247-22. doi: 10.1128/msystems.00247-22 (PMC9426475; doi:10.1128/msystems.00247-22)
Supplement: TABLE S1 [file msystems.00247-22-s0004.docx]

**Table S1**.

| **Step 1: 1μl of template DNA** |  |  |
| --- | --- | --- |
| **Reagent** | **Quantity (μl)** | **Reaction conditions:** |
| 10X buffer | 2.5 | 95℃ for 5.00 min; 30 cycles at 95℃ for 30s, 55℃ for 30s, 72℃ for 1min; 72℃ for 5 min; 4℃ forever. |
| MgCl_2_ (25mM) | 0.5 |  |
| 515F (10uM) / ITS1F (10uM) | 0.5 |  |
| 806R (10uM) / 58A2R (10uM) | 0.5 |  |
| dNTP (10 mM) | 0.5 |  |
| Taq (5U/μl) | 0.125 |  |
| BSA (50 mg/ml) | 0.25 |  |
| Filter Nuclease-Free H_2_O | Fill to final 25 |  |
|  |  |  |
| **Step 2: 5μl of DNA product from step 1** |  |  |
| **Reagent** | **Quantity (μl)** | **Reaction conditions:** |
| 10X buffer | 2.5 | 95℃ for 5.00 min; 8 cycles at 95℃ for 30s, 55℃ for 30s, 68℃ for 30s; 68℃ for 5 min; 4℃ forever. |
| MgCl_2_ (25mM) | 0.5 |  |
| Index primerF (5uM) | 2 |  |
| Index primerR (5uM) | 2 |  |
| dNTP (10 mM) | 0.5 |  |
| Taq (5U/μl) | 0.125 |  |
| BSA (50 mg/ml) | 0.25 |  |
| Filter Nuclease-Free H_2_O | Fill to final 25 |  |
